# An “unclassified diabetes” after a long duration of disease in a young subject: a case report

**DOI:** 10.11604/pamj.2025.50.16.46217

**Published:** 2025-01-07

**Authors:** Mesmin Dehayem Yefou, Jean Claude Katte, Anne Ongmeb Boli, Martine Claude Etoa Etoga, Laeticia Josseline Yongoua Kouayep, Eugène Sobngwi

**Affiliations:** 1 Department of Internal Medicine and Specialties, Faculty of Medicine and Biomedical Sciences, University of Yaoundé I, Yaoundé, Cameroon,; 2 Endocrine and Diabetology Service, Yaoundé Central Hospital, Yaoundé, Cameroon,; 3 Department of Clinical and Biomedical Sciences, University of Exeter Medical School, Exeter, United Kingdom,; 4 Faculty of Health Sciences, University of Bamenda, Bamenda, Cameroon

**Keywords:** Unclassified diabetes, type 1 diabetes, long duration, diagnosis, young subject, case report

## Abstract

Unclassified diabetes describes diabetes that does not fit into other categories. This category is used temporarily when there is no clear diagnostic category, especially close to the time of diagnosis. This implies that paraclinical investigations or the natural evolution of the disease will allow a definitive classification. We present the case of diabetes initially classified as type 1 in an adolescent. For ten years, the patient had high insulin requirements with negative autoantibodies, significant residual insulin secretion, and for the last 3 months of life, remission of diabetes in the context of a wound of the thigh, before a sudden death from hepatocellular failure, pushing to reconsider the initial diagnosis. This clinical case highlights difficulties in obtaining precision in the etiological diagnosis of diabetes in some patients based either on clinical manifestation, biological assessment, or evolution. It also shows that unclassified diabetes subtypes may not be transient.

## Introduction

Diabetes is a group of heterogeneous metabolic disorders characterized by chronic hyperglycemia because of insulin resistance and/or relative insulin deficiency, leading to end-organ damage and complications [1]. Etiological diagnosis of diabetes is particularly important for precision diabetes treatment [2,3]. However, a wide heterogeneity in the phenotypes of all diabetes subtypes, including type 1 diabetes, type 2 diabetes, and monogenic diabetes, has been reported and contributes to frequent misdiagnosis [2]. It is in this context that World Health Organization (WHO) in its 2019 revised classification of diabetes introduced two new categories including the “unclassified diabetes” category [1]. This category is used temporarily when there is no clear diagnostic category, especially close to the time of diagnosis. This category of diabetes which upon diagnosis cannot be considered type 1, type 2, or secondary diabetes, is also called atypical diabetes [3]. Many cases of atypical diabetes now have a known etiology and are monogenic diabetes [3]. We report on a case of a young person who had diabetes as an adolescent and whose initial clinical picture was that of type 1 diabetes. However, the paraclinical examination that was subsequently done, the clinical evolution of diabetes as well as the condition of death that occurred ten years after, pushed us to reconsider the initial diagnosis without having a precise idea of the etiology of diabetes.

## Patient and observation

**Patient information:** in June 2013, a 17-year-old male Cameroonian teenager was brought to consultation at the Endocrine and Diabetology Service of the Yaoundé Central Hospital, Yaoundé, Cameroon, for polyuria, polydipsia, unquantified weight loss and asthenia evolving for approximately 3 months. His mother was deceased, and his father was still alive working as a carpenter. There was no parental or first-degree-relative history of diabetes.

**Clinical findings:** upon physical examination, the patient had normal vital signs, with a weight of 65 kg, height of 1.68 m, body mass index of 23 kg/m^2^, and an abdominal circumference of 73 cm. The rest of the physical examination was unremarkable.

**Timeline:** until his admission at the Yaoundé Central Hospital, the patient had not yet consulted in any other health facility for the same symptoms.

**Diagnostic evaluation:** the capillary blood glucose was > 600 mg/dL, ketonuria negative and the Hemoglobin A1C (HbA1c) was 11.7%.

**Diagnosis:** the diagnosis of hyperglycemic crisis in a teenager with type 1 diabetes was made.

**Therapeutic intervention:** after a brief hospitalization during which he received appropriate care including therapeutic education, he was discharged from the hospital on human insulins (regular insulin and neutral protamine Hagedorn) 30 units per day divided into three injections. He also received a glucometer and strips for monitoring diabetes. This treatment was free after enrollment in the “Changing Diabetes in Children” project in Cameroon [4].

**Follow-up and outcome:** he came into the hospital every 1-3 months for the follow-up or the collection of material for the treatment. When an assessment was done after 6 months of treatment, he weighed 72 kg, HbA1c was 6.1%, and there was no episode of severe hypoglycemia. HbA1c remained within the target (< 7%) for the first 3 years, then rose and varied between 8 and 9% for the following 7 years, during quarterly or six-monthly visits, giving a ten-year average of 7.8 ± 1.6% (5.5-11.7%) (Table 1). During this period, the average weight was 72 ± 2 kg (65-75), and the average daily insulin dose was 39 ± 8 units (32-58). In 2019, six years after the diagnosis of diabetes, the etiological assessment demonstrated a residual insulin secretion with random non-fasting C-peptide at 222 pmol/L (reference range > 200) and the absence of islet autoantibodies. Anti-glutamic acid decarboxylase (GAD65) was at 3.058 IU/mL (reference range < 11), anti-tyrosine phosphatase islet antigen 2 (IA-2) was at 4.977 IU/mL (reference range < 7.5) and anti-zinc transporter 8 (ZnT8) was at 5.108 IU/mL (reference range < 65). Type 1 diabetes genetic susceptibility assessed using a 67 single nucleotide polymorphism (SNP) genetic risk score (T1DGRS) was at 5.31 (a value less than the 50th percentile for a normal Cameroonian population without diabetes), and the corresponding imputed Human Leukocyte Antigen (HLA) revealed non-DR3/non-DR4. In June 2022, he had cellulitis of the lower left limb. After being treated in a surgical department, he had a large wound in his left thigh and leg, for which he regularly went to a health center for dressings. In December 2022, while the wound, although not infected, was still very large, he began to have episodes of hypoglycemia.

**Table 1:** HbA1c, fasting capillary blood glucose, weight and insulin doses during quarterly or annual routine visits of the patient

Date	June 2013	December 2013	June 2014	December 2014	June 2015	June 2016	September 2016	December 2016	December 2017	December 2018	June 2019	December 2019	March 2022
HbA1c (%)	11.7	6.1	5.5	5.6	7.0	6.9	9.0	8.5	9.3	8.2	8.2	9.4	8.3
Fasting blood glucose (mg/dL)	600	118	109	158	252	239	400	330	-	257	-	186	267
Weight (kg)	65	72	74	71	72	74	75	73	73	72	72	72	70
Insulin doses (Units/24hours)	30	38	-	32	32	46	40	46	38	40	-	32	58

The HbA1c was assessed between 2013-2015 by the In2itTM Point-of-care system (Bio-Rad Laboratories, Deeside, UK), between 2016-2020 by the D-10 (Bio-Rad Laboratories, Hercules, United States), and since 2021 by the point-of-care system HemoCue® HbA1c 501. (-) represents a missing data in the file of the patient. After being consistent with his quarterly and annual visits at the Children Diabetes Clinic of the Yaoundé Central Hospital, Yaoundé, Cameroon; between 2013 and 2016, the patient reduced his visits from 2017. In 2020 and 2021, he did not come at all; HbA1c: Hemoglobin A1C

Despite a gradual reduction in insulin doses, the persistence of hypoglycemia led to insulin cessation. In February 2023, he was hospitalized in a state of anasarca. Apart from the known large wound on the left limb, there were lesions of epidermolysis on some parts of his body. The body temperature was 37.2°C, the blood pressure at 131/96 mm Hg, the pulse rate at 100 beats/minute, and the respiratory rate at 20 breaths per minute. Fasting capillary blood glucose was 108 mg/L, white blood cells at 6700/mm^3^, hemoglobin level at 8.7g/dL, MCV at 69 fL, CRP at 173mg/L, prothrombin rate at 47.7%, serum albumin level at 8.4g/L, ALAT at 135 IU/L, ASAT at 121 IU/L, anti-HCV antibody negative, hepatitis B surface antigen (HBsAg) negative, serum creatinine at 11mg/L, blood urea at 0.35g/L, proteinuria negative, sodium at 137 meq/L, potassium at 5.1 meq/L, chloride at 110 meq/L, and calcemia (ionized) at 53mg/L. A liver failure secondary to the consumption of some herbal products was suspected. The patient was treated with diuretics, a noncaloric protein diet, and skin care. His general condition improved. However, after 8 days of admission, he developed jaundice, then hepatic encephalopathy, and died within 48 hours. Throughout his stay in the hospital and despite the cessation of insulin therapy in the last 3 months, the pre and postprandial blood glucose levels were normal.

**Informed consent:** written informed consent was obtained from the patient´s father prior to enrollment in the “Changing Diabetes in Children” project, authorizing the use of data from his medical record for research and publication purposes.

## Discussion

We have reported on a case of diabetes in a young male subject diagnosed as having type 1 diabetes a decade ago, with etiologic assessment showing the absence of islet autoantibody markers, significant residual insulin secretion, who had a remission of diabetes for three months after infectious cellulitis of the thigh before dying suddenly of hepatocellular insufficiency of unknown origin. This case highlights the difficulty that can occur in making the precise diagnosis of diabetes, especially in limited resort settings. Although the diagnosis of type 1 diabetes was initially made based on clinical manifestations, the absence of islet autoantibodies, the persistence of significant residual insulin secretion over a long period, and the remission ten years after the diagnosis led to reconsidering this diagnosis. Type 1 autoimmune diabetes is usually defined by a near-complete deficiency in insulin secretion due to the autoimmune destruction of beta cells, which is associated with the presence of anti-beta cell autoimmunity markers, particularly the autoantibodies directed to insulin, GAD, IA-2, and ZnT8. Type 1 diabetes occurs in the context of genetic susceptibility, mainly defined by certain human leukocyte antigen (HLA) class II alleles [2]. The absence of islet autoantibodies is not uncommon in long-duration diabetes, since 5-10% of people with type 1 diabetes do not have autoantibodies [5], but the absence of hallmark type 1 diabetes susceptibility HLA markers of DR3 and/or DR4 further makes the diagnosis of type 1 diabetes less likely in this case. However, it is unusual to have persistent significant insulin secretion in long-standing diabetes.

The absence of insulinopenia and episodes of ketosis did not allow classification as idiopathic type 1 diabetes [6]. Type 2 diabetes was unlikely due to age diagnosis, lack of weight, and family history of diabetes. Three monogenic diabetes that are associated with liver disease could be mentioned. The first is lipoatrophic diabetes which can lead to end-stage liver disease secondary to nonalcoholic steatohepatitis [7]. However, our patient did not have a paucity of fat or insulin resistance to suggest lipoatrophic diabetes. The association between Maturity-onset diabetes of the young type 3 (MODY-3) and liver adenomatosis has been reported [8]. However, liver adenomatosis does not lead directly to end-stage liver disease. The association between MODY-5 and an increased liver enzyme has been reported [9]. Candidates for genetic testing for MODY include nonobese subjects with hyperglycemia, no evidence of Î^2^-cell autoimmunity, preserved Î^2^-cell function, and a strong family history of similar-type diabetes among first-degree relatives [10]. Although this patient had many criteria in favor of, the probability of MODY seemed low in the absence of a family history of similar diabetes in first-degree relatives and high insulin requirements. Because his clinical profile did not fit with one of the known MODY types, genetic testing was not performed given the cost and logistic constraints associated with testing.

There are some limitations in this case report. Having performed autoantibody screening six years after the diagnosis of diabetes was a bias concerning their absence since the frequency of autoantibodies is higher close to diagnosis and their titers diminish with time [1]. When diabetes remission occurred, the patient was followed in the surgery department for a wound of the thigh and joined the diabetology department only after the development of hepatocellular failure. Hence, the exploration of this remission was incomplete. The sudden death of this patient from liver failure also complicated explorations in a context where it was impossible to carry out an autopsy without family consent. This is therefore a form of diabetes perfectly mimicking type 1 diabetes at diagnosis and during the first years of disease, but which is differentiated by the absence of islet autoantibodies and significant residual insulin secretion for a decade allowing insulin withdrawal for the last months of the patient's life without hyperglycemia and ketosis. The fatal evolution after 10 years in the context of complications not directly related to diabetes but in association with liver failure (although the toxic origin was suspected) is quite atypical. Even if the explorations were insufficient, this clinical case clearly corresponds to a type of diabetes that does not correspond to any already described form in the literature

## Conclusion

This case reveals that unclassified diabetes which has now been recognized as a true diabetes category by the most recent WHO diabetes classification is real. It also highlights the complexity of making a precise diagnosis of diabetes and how long-term follow-up and detailed etiologic assessment can be important in reviewing the classification of diabetes.
